# Psychotic spectrum symptoms across the lifespan are related to lifetime suicidality among 147 patients with bipolar I or major depressive disorder

**DOI:** 10.1186/s12991-016-0101-7

**Published:** 2016-06-21

**Authors:** Camilla Gesi, Claudia Carmassi, Mario Miniati, Antonella Benvenuti, Gabriele Massimetti, Liliana Dell’Osso

**Affiliations:** Department of Clinical and Experimental Medicine, University of Pisa, Via Roma 67, 56100 Pisa, Italy

**Keywords:** Mood disorders, Bipolar I disorder, Major depressive disorder, Psychotic spectrum, Suicidality

## Abstract

**Background:**

Conflicting evidence exists about the relationship between psychotic symptoms and suicidality in mood disorders. We aimed to investigate the lifetime suicidality and its relationship with dimensions of the psychotic spectrum over the lifespan among subjects with bipolar I (BD I) or major depressive disorder (MDD).

**Methods:**

147 Consecutive out- and inpatients with BD I or MDD presenting for treatment at 11 Italian Departments of Psychiatry were administered the Structured Clinical Interview for DSM-IV Axis I Disorders, the Structured Clinical Interview for the Psychotic Spectrum (SCI-PSY, *lifetime version*) and the Mood Spectrum Self-Report (MOODS-SR, *lifetime version*).

**Results:**

Subjects with psychotic features did not differ from those without for MOODS-SR suicidality score. Controlling for age, gender and diagnosis (MDD/BD I), the SCI-PSY total score (*p* = .007) and *Paranoid* (*p* = .042), *Schizoid* (*p* = .007) and *Interpersonal Sensitivity* (*p* < .001) domain scores independently predicted lifetime MOODS-SR suicidality score in the overall sample.

**Conclusions:**

Psychotic features, as evaluated upon the presence of delusions or hallucinations, are not associated with suicidality among subjects with BD I or MDD. However, more subtle dimensions of the psychotic spectrum, such as *Interpersonal Sensitivity, Paranoid* and *Schizoid* symptoms, show a significant relationship with lifetime suicidality. Our findings highlight the potential usefulness of a spectrum approach in the assessment of psychotic symptoms and suicide risk among subjects with BD I or MDD.

## Background

Suicide is one of the leading causes of preventable death, with more than 800,000 people dying by self-inflicted injuries every year [1].

In high-income countries, up to 90 % of people who die by suicide suffer from a mental disorder [2]. In particular, mood disorders are among the most important risk factors for suicidality [3–6]. The lifetime risk for suicide is estimated to be around 4 % in patients with any mood disorder [7] and 8 % in people with bipolar disorder [1, 8, 9]. Importantly, the risk of suicidal behavior increases with comorbidity. Significantly higher risk of suicide has been in fact reported among individuals with more than one mental disorder, with odds of serious suicide attempts 89.7 times the odds of those with no psychiatric disorders [2, 10]. Moreover, recent data suggest that also subthreshold comorbidity might increase the risk of suicidal ideation and attempts [11–15].

Several investigations in clinical and non-clinical samples have evaluated the relationship between psychotic symptoms and the risk of developing suicidal thoughts or behaviors. Recent epidemiological data have shown subthreshold psychotic experiences to be predictive of suicidal thoughts and attempts in non-clinical populations [16–23]. Moreover, a number of studies have identified a correlation between positive psychotic symptoms, or positive psychotic symptoms-related distress, and suicide in patients with schizophrenia [24–26]. As for mood disorders, some authors suggested that suicidal behaviors might be more frequent in patients with mood disorders with psychotic features with respect to those without [27], despite this has not been confirmed in all studies [28, 29]. Ran and colleagues [30] found higher levels of delusions in suicide attempters than in those without suicide attempts among 166 patients with affective disorders. Park et al. [31] reported the same result among 1183 subjects with depression. Suicidal ideation was found to be associated with psychotic features among 644 subjects with unipolar or bipolar II major depression [32]. Song and colleagues [33] found auditory hallucinations and the age of first psychotic symptoms to be significant predictors of suicide attempts among 212 subjects with bipolar disorder. Conversely, Pawlak and colleagues [34] failed to find an association between psychotic symptoms and suicide attempts among 597 subjects with unipolar and bipolar affective disorders. Consistently, in a recent register-based, nationwide, historical prospective cohort study, evaluating 34,672 severely depressed subjects followed at Danish psychiatric hospitals between 1994 and 2010, psychotic depression was not identified as an independent risk factor for suicide [35].

However, the extensive literature on suicide risk factors among subjects with mood disorders has mostly been focused on positive psychotic symptoms, while more subtle dimensions of the psychotic spectrum have not been hitherto carefully investigated.

*Aim of the study* The aim of this paper is to investigate lifetime suicidality in a sample of patients with bipolar I (BD I) or major depressive disorder (MDD), either with or without psychotic features, and to which dimensions of the psychotic spectrum that may have been present during the entire lifespan is associated.

## Methods

The data were collected in 11 Italian Departments of Psychiatry located at nine sites: Pisa, Bari, Cagliari, Firenze, Milano, Sassari, Siena, Torino, and Udine.

### Participants

A consecutive sample of out- and inpatients presenting for treatment at the 11 Italian Departments of Psychiatry were invited to participate in the study. Eligible patients included new and continuing patients between 18 and 60 years of age diagnosed with BD I or MDD. Exclusion criteria were severe medical illness or neurological diseases with a known etiological link to mood disorders (e.g., malignancies, severe autoimmune conditions, degenerative neurological diseases, stroke) current substance use disorder and inability to participate because of the severity of psychiatric symptoms.

### Ethics, consent and permissions

The Ethics Committee of the Azienda Ospedaliero-Universitaria of Pisa (Italy) approved all recruitment and assessment procedures. Eligible subjects provided written informed consent, after receiving a complete description of the study and having an opportunity to ask questions. Subjects were not paid for their participation.

### Measures

Participants filled the Mood Spectrum Self-Report [36] (MOODS-SR, *lifetime version*) and were administered the Structured Clinical Interview for DSM-IV Axis I Disorders (SCID-I/P) [37] and the Structured Clinical Interview for the Psychotic Spectrum (SCI-PSY, *lifetime version*) [38] through face-to-face interview. The assessment was conducted by psychiatrists or residents in psychiatry, who were trained and certified in the use of the study instruments at the Department of Psychiatry of the University of Pisa.

#### The Structured Clinical Interview for the Psychotic Spectrum (SCI-PSY, *lifetime version*)

The SCI-PSY is a 164-item questionnaire assessing a broad range of psychopathological manifestations associated with psychotic syndromes or schizoid/schizotypal personality disorders, as well as atypical and subthreshold psychotic symptoms, occurring during the lifetime of an individual [38]. The SCI-PSY items are organized into five domains. The *Interpersonal Sensitivity* domain describes the tendency to avoid others due to the fear of being misunderstood or criticized or to vague perceptions of hostile attitudes of the others. The *Paranoid* domain covers the spectrum features encompassing mild hypervigilance, diffidence, suspiciousness, interpretive attitude and paranoid self-reference. The *Schizoid* domain explores lack of emotional resonance, difficulty in showing feelings, being unemotional and unromantic, the tendency to be isolated, which is typical of autism and schizoidism, and religiosity, superstition, magical and odd thoughts. The *Misperceptions* domain explores the presence of dissociative phenomena with partial or no insight, such as experiencing sudden changes in the environment, without being able to specify which ones; feeling that everything around is changing and becoming unfamiliar and unreal, perceiving sounds as unbearable and amplified, or recognizing in the background indistinguishable voices, or feeling the body or the reality as suddenly changed or strange. The *Typical psychotic symptoms* domain includes the psychotic symptoms listed in the DSM-IV such as delusions (e.g., persecution, reference, guilt, ruin, megalomanic and erotomanic), auditory hallucinations (e.g., noises or whispering voices or voices talking together) and visual and somatic hallucinations.

#### Mood Spectrum Self-Report (MOODS-SR, *lifetime version*)

The MOODS-SR questionnaire is focused on the presence of manic and depressive symptoms, traits and lifestyles that may characterize the ‘temperamental’ affective dysregulation that make up both fully syndromal and sub-threshold mood disturbances. The latter includes symptoms that are either isolated or clustered in time and temperamental traits that are present throughout an individual’s lifetime. The MOODS-SR consists of 161 items coded as present or absent for one or more periods of at least 3–5 days in the lifetime. Items are organized into three manic–hypomanic and three depressive domains each exploring mood, energy and cognition, plus a domain that explores disturbances in rhythmicity and in vegetative functions, including sleep, appetite and sexual function. The sum of the scores on the three manic–hypomanic domains constitutes the score for the manic–hypomanic component and that on the three depressive domains the depressive component.

### Assessment of suicidality

Suicidality was assessed by means of six items of the MOODS-SR that explore whether the subject has ever experienced periods of 3–5 days or more when he or she thought that life was not worth living (*N* = 102); wished he/she would not wake up in the morning, or that he/she would die in an accident or from something like a heart attack or a stroke (*N* = 103); wanted to die or hurt him/herself (*N* = 104); wanted to die and had a specific plan to hurt or kill him/herself (*N* = 105); actually committed a suicide attempt (*N* = 106); committed a suicide attempt that required medical attention (*N* = 107). For the purpose of this study, suicidality was scored according to the number of positive answers given on these six items by each patient, retrieving a score ranging from 0 to 6.

### Statistical analyses

Chi-square and *t* tests were used to compare demographical and clinical variables between groups. Multivariate linear regressions were undertaken to examine whether SCI-PSY total and subscale scores predicted suicidality. Analyses were conducted using SPSS, version 20.0 [39].

## Results

### Sample characteristics

Suicidality data were missing in two (1.3 %) patients out of the total sample of 147 subjects; after the exclusion of these 2 participants, the final sample of 145 subjects comprised: 59 with BD I (35 with psychotic features according to DSM-IV, 24 with no psychotic features); 86 with MDD (18 with psychotic features, 68 with no psychotic features).

Subjects with BD I and MDD did not differ in age (38.3 ± 10.8 vs 41.9 ± 11.5; *t* = 1.911; *p* = .058). The demographic and clinical characteristics of the study sample are provided in Table 1. Subjects with BD I and MDD did not differ in the MOODS-SR suicidality score (*t* = .752; *p* = .454) or for all SCI-PSY domain scores but in *Typical Symptoms* (*t* = −4.005; *p* = **<**.001).

**Table 1:** Characteristics of the study sample

	MDD (*n* = 86)	BD I (*n* = 59)	*χ* ^2^	*p*
	*N* (%*)	*N* (%*)		
Female	60 (69.8)	31 (52.5)	4.442	*.038*
Male	26 (30.2)	28 (47.5)		
Marital status				
Not married	57 (66.3)	42 (72.4)	.436	.469
Married	29 (33.7)	16 (27.6)		
Education				
Graduate/post-graduate	14 (16.3)	10 (17.2)	1.478	.687
High school	27 (31.4)	23 (39.7)		
Secondary school	34 (39.5)	20 (34.5)		
Elementary school	11 (12.8)	5 (8.6)		
Occupation				
Unoccupied	50 (58.1)	34 (59.6)	1.000	.498
Occupied	36 (41.9)	23 (40.4)		
Current acute phase	32	23	.865	.477
Treatment status				
Pre-treatment	6 (7.0)	6 (10.2)	2.884	.410
Acute-phase treatment	36 (41.9)	19 (32.2)		
Maintenance	41 (47.7)	29 (49.2)		
Follow-up	3 (3.5)	5 (8.5)		

	*mean* *±* *SD*	*mean* *±* *SD*	*t* test	*p*
MOODS-SR lifetime				
Total	57.2 (23.4)	73.2 (26.2)	3.868	*<.001*
*Depressive component*	32.2 (14.1)	32.4 (14.6)	.067	.946
*Manic component*	13.9 (10.9)	28.7 (12.0)	7.689	*<.001*
*Lifetime suicidality*	2.4 (2.2)	2.3 (2.2)	.752	.454
SCI-PSY lifetime				
Total	42.5 (21.9)	49.2 (23.3)	1.777	.078
*Interpersonal Sensitivity*	4.5 (2.7)	4.1 (2.7)	−.953	.342
*Paranoid*	27.4 (14.7)	30.0 (15.2)	1.047	.297
*Schizoid*	6.3 (3.2)	6.5 (3.6)	.419	.676
*Misperceptions*	1.3 (1.5)	1.8 (1.9)	1.763	.081
*Typical symptoms*	3.1 (3.2)	6.8 (6.8)	4.005	*<.001*

Italic values indicate statistically significant (<0.05)

* Column percentages

### Relationship between suicidality and psychotic features/SCI-PSY subscales

Subjects with psychotic features did not differ from those without for MOODS-SR suicidality score (*t* = .877; *p* = .382). Linear regression was used with MOODS-SR suicidality score as the dependent variable. After controlling for the effects of age, gender and diagnosis (MDD/BD I), a series of regression analyses were conducted. The total SCI-PSY score and each subscale were individually inserted in the second block to determine whether different dimensions of the psychotic spectrum significantly predicted lifetime suicidality. The results showed that the SCI-PSY total score (*β* = .228; *p* = .007), as well as the *Interpersonal Sensitivity* (*β* = .292; *p* < .001), *Paranoid* (*β* = .172; *p* = .042) and *Schizoid* (*β* = .228; *p* = .007) domains scores independently predicted lifetime MOODS-SR suicidality score in the overall sample. After only significant domains were inserted in a multivariate regression analysis, also including the total depressive mood score of the MOODS-SR, the correlation remained significant only for the *Interpersonal Sensitivity* (*B* = .164; *p* = .044) subscale score (Table 2).

**Table 2:** Relationship between SCI-PSY subscales and MOODS-SR suicidality score in the overall sample

	*B* (SE)	*β*	95 % CI	*p*	R^2^
Block 1					
Age	−.007 (.016)	−.038	−.039–.025	.657	.014
Sex	.431 (.377)	.097	−.313–1.176	.254	
Diagnosis (MDD/BD)	.322 (.375)	.074	−.419–1.062	.392	
Block 2					
*Interpersonal sensitivity*	.164 (.081)	.203	.004–.324	*.044*	.303
*Paranoid*	−.023 (.016)	−.157	−.054–.009	.154	
*Schizoid*	.098 (.058)	.153	−.017–.213	.095	
*MOODS*-*SR depressive mood*	.154 (.025)	.461	.104–.205	*<.001*	

The model entered age, sex and diagnosis (MDD/BD) in block 1 and SCI-PSY subscales, together with total depressive mood scores of the MOODS-SR in block 2

Italic values indicate statistically significant (<0.05)

## Discussion

To the best of our knowledge, this is the first study investigating the relationship between psychotic symptoms over the lifespan and lifetime suicidality among individuals with BD I or MDD by means of a spectrum approach. The majority of studies, in fact, explored the prevalence of suicidal thoughts or attempts comparing patients with the presence of psychotic symptoms such as delusions and hallucinations, retrieving conflicting evidences [32–35].

Our results show no differences in lifetime suicidality between subjects with MDD and those with BD I. Moreover, we did not find a significant correlation between suicidality and the presence of psychotic features. According to DSM-IV criteria, these latter were assessed on the presence of delusion and/or hallucinations. However, evaluating the study sample by means of our spectrum instrument encompassing not only typical positive symptoms, such as hallucinations and delusions, but also atypical, subthreshold and negative manifestations, our results suggest that further dimensions of the psychotic spectrum occurring across the lifespan may have a significant relationship with lifetime suicidality. In particular, linear regression analyses indicated lifetime suicidality to be significantly related to the *Paranoid,* the *Schizoid* and the *Interpersonal Sensitivity* domain score of the SCI-PSY. The latter domain remained significantly correlated to lifetime suicidality even when inserted in a regression model that included the other SCI-PSY domains correlated to suicidality and the total depressive lifetime score of the MOODS-SR. While the final regression model showed to account only for a moderate percentage of variance in suicidality (about 30 %), the results showed that for one unit increase in *Interpersonal Sensitivity*, suicidality is expected to increase by .164 units.

Some previous studies reported a link between suicidality and interpersonal sensitivity. Interpersonal sensitivity has been found to discriminate between individuals engaging in self-harm and those who do not engage in such behaviors among 1986 Air Force recruits [40]. Engin et al. [41] found interpersonal sensitivity among the risk factors for suicidal thoughts in a sample of 1992 first-year university students. Consistently, Gupta et al. [42] reported that interpersonal sensitivity might represent the mediating factor between cutaneous body image dissatisfaction and suicidal ideation in a non-clinical sample of 312 subjects. As for clinical samples, Stepp et al. [43] evaluated the relationship between attachment styles and self-injury among 406 patients with a broad range of mental disorders, including both Axis I and Axis II disorders, highlighting that this relationship is arguably mediated by interpersonal sensitivity. We may argue that this latter may render individuals prone to feel easily hurt or rejected, and thus to avoid approaching others, having pessimistic expectancies about the outcome of their interpersonal interactions; as a consequence, they may experience feelings of personal inadequacy and negative emotional states, such as depression or anxiety. In addition, such avoidance may worsen social isolation and social exclusion that have been shown, in turn, to be associated with increased suicidal risk [44–46].

Our data also showed a relationship between the *Paranoid* domain of the SCI-PSY and lifetime suicidality. An increase in paranoid behaviors has been indicated as a signal of risk of suicide among subjects with schizophrenia [47]. Consistently, the paranoid schizophrenia subtype has been repeatedly associated with an elevated risk of suicide among other types of schizophrenia [48, 49]. As far as we know, however, the relationship between paranoid symptoms and suicidality has not been specifically evaluated among subjects with BD I or MDD.

Further, we found a relationship between lifetime suicidality and the *Schizoid* domain of the SCI-PSY. This domain explores the lifetime occurrence of symptoms that are typical of autism, schizoidism and schizotypy. The comorbidity with cluster A personality disorders has been repeatedly shown to increase the risk for suicide in patients with different mental disorders, including mood disorders [50–52]. On the other hand, growing data indicate that autistic traits may both represent an independent risk factor for suicide and increase the likelihood of engaging in more lethal suicidal behaviors in depressed patients [53, 54]. Such literature also appears consistent with recent reports indicating that subjects committing almost fatal suicide attempts show significantly lower self-disclosure, more schizoid tendencies and more feelings of loneliness and communication difficulties than those who did not report medically serious consequences [55–57]. We might speculate that subjects with autism spectrum symptoms and those with schizoid or schizotypal traits share a common experience of interpersonal problems, lack of intimacy and social isolation [12]. Such symptoms are likely to lead to difficulties in accessing social support and to reduce the individual’s resilience to stressful life events [58], increasing feelings of helplessness and hopelessness and, ultimately, giving rise to suicidal thoughts and behaviors.

Several limitations of this study have to be acknowledged while interpreting our findings. First, we grouped together patients from both in- and outpatient treatment settings, which may have biased reports of lifetime suicidality. Second, the lack of information about age of onset of mood disorder, number of episodes, current treatment and the lack of assessment of Axis II comorbidity impaired us from analyzing the possible contribution of these factors to psychotic spectrum symptoms. Further, suicidality was assessed solely by selecting six questions of a 161-item instrument and grouping together very different aspects, from ideas that life is not worth living up to attempted suicide, preventing us from elucidating whether some dimensions of the psychotic spectrum specifically correlate with suicidal behaviors rather than with transient suicidal thoughts. Moreover, the use of a lifetime assessment did not allow us to establish whether the psychotic symptoms preceded, co-occurred or followed suicidal ideation or attempts. Last, the exclusion of individuals with severe medical and neurological disorders may have resulted in an underestimation of lifetime suicidality in patients with BD I or MDD.

## Conclusions

In the context of the limitations listed above, we are reporting a significant association between lifetime suicidality and some dimensions of the psychotic spectrum occurring over the lifespan among patients with BD I or MDD. Our findings suggest the potential usefulness of a spectrum approach in the assessment of suicidality among patients with mood disorders. On the other hand, they highlight the importance of a dimensional approach to rate severity for the core symptoms of schizophrenia not only among patients with schizophrenia spectrum and other psychotic disorders [59], but also among those with mood disorders. Further research is needed to corroborate our results and to elucidate what implications, if any, the findings have on the assessment and treatment of patients with BD I or MDD.

## Abbreviations

SCI-PSY: Structured Clinical Interview for the Psychotic Spectrum
MOODS-SR: Mood Spectrum Self-Report
PM: Psychotic mood disorders
NPM: Non-psychotic mood disorders
